# Supplementary material for: The Nasopharyngeal, Ruminal, and Vaginal Microbiota and the Core Taxa Shared across These Microbiomes in Virgin Yearling Heifers Exposed to Divergent In Utero Nutrition during Their First Trimester of Gestation and in Pregnant Beef Heifers in Response to Mineral Supplementation
Source: Microorganisms. 2021 Sep 23;9(10):2011. doi: 10.3390/microorganisms9102011 (PMC8537542; doi:10.3390/microorganisms9102011)
Supplement: Supplementary file 1 [file microorganisms-09-02011-s001.zip › microorganisms-1367805-supplementary.pdf]

Supplementary Table S1. OTUs identified in the nasopharyngeal, ruminal and vaginal microbiota of at least 60% of samples from virgin yearling heifers.

OTU	Taxa	60%	65%	70%	75%	80%	85%	90%	95%	100%
OTU8	[k__Archaea, p__Euryarchaeota, c__Methanobacteria, o__Methanobacteriales, f__Methanobacteriaceae, g__Methanobrevibacter, s__NA]	■	■	■						
OTU23	[k__Archaea, p__Euryarchaeota', c__Methanobacteria, o__Methanobacteriales, f__Methanobacteriaceae, g__Methanobrevibacter, s__ruminantium]	■	■	■	■	■				
OTU68	[k__Bacteria, p__Actinobacteriota, c__Actinobacteria, o__Bifidobacteriales, f__Bifidobacteriaceae, g__Bifidobacterium, s__pseudolongum]	■								
OTU147	[k__Bacteria, p__Actinobacteriota, c__Actinobacteria, o__Corynebacteriales, f__Corynebacteriaceae, g__Corynebacterium, s__marinum]	■								
OTU26	[k__Bacteria, p__Actinobacteriota, c__Actinobacteria, o__Corynebacteriales, f__Corynebacteriaceae, g__Corynebacterium, s__NA]	■	■	■	■	■				
OTU160	[k__Bacteria, p__Actinobacteriota, c__Actinobacteria, o__Micrococcales, f__Intrasporangiaceae, g__Ornithinimicrobium, s__NA]	■								
OTU35	[k__Bacteria, p__Actinobacteriota, c__Actinobacteria, o__Streptomycetales, f__Streptomycetaceae, g__Streptomyces, s__NA]	■								
OTU351	[k__Bacteria, p__Actinobacteriota, c__Coriobacteriia, o__Coriobacteriales, f__Atopobiaceae, g__Atopobium, s__NA]	■								
OTU368	[k__Bacteria, p__Actinobacteriota, c__Coriobacteriia, o__Coriobacteriales, f__Eggerthellaceae, g__DNF00809, s__NA]	■								
OTU392	[k__Bacteria, p__Firmicutes, c__Clostridia, o__Christensenellales, f__Christensenellaceae, g__Christensenellaceae R-7 group, s__NA]	■								
OTU537	[k__Bacteria, p__Firmicutes, c__Clostridia, o__Christensenellales, f__Christensenellaceae, g__Christensenellaceae R-7 group, s__NA]	■								
OTU927	[k__Bacteria, p__Firmicutes, c__Clostridia, o__Clostridia UCG-014, f__NA, g__NA, s__NA]	■								
OTU97	[k__Bacteria, p__Firmicutes, c__Clostridia, o__Hungateiclostridiaceae, f__Saccharofermentans, g__NA, s__NA]	■	■	■	■					
OTU133	[k__Bacteria, p__Firmicutes, c__Clostridia, o__Lachnospirales, f__Lachnospiraceae, g__[Eubacterium] hallii group, s__NA]	■	■	■	■	■				
OTU1335	[k__Bacteria, p__Firmicutes, c__Clostridia, o__Lachnospirales, f__Lachnospiraceae, g__[Eubacterium] hallii group, s__NA]	■								
OTU158	[k__Bacteria, p__Firmicutes, c__Clostridia, o__Lachnospirales, f__Lachnospiraceae, g__[Eubacterium] hallii group, s__NA]	■	■							
OTU3351	[k__Bacteria, p__Firmicutes, c__Clostridia, o__Lachnospirales, f__Lachnospiraceae, g__[Eubacterium] hallii group, s__NA]	■	■							
OTU489	[k__Bacteria, p__Firmicutes, c__Clostridia, o__Lachnospirales, f__Lachnospiraceae, g__[Eubacterium] hallii group, s__NA]	■								
OTU1111	[k__Bacteria, p__Firmicutes, c__Clostridia, o__Lachnospirales, f__Lachnospiraceae, g__Coprococcus, s__NA]	■		■						
OTU1688	[k__Bacteria, p__Firmicutes, c__Clostridia, o__Lachnospirales, f__Lachnospiraceae, g__Lachnospiraceae NK3A20 group, s__NA]	■	■	■	■	■				
OTU1742	[k__Bacteria, p__Firmicutes, c__Clostridia, o__Lachnospirales, f__Lachnospiraceae, g__Lachnospiraceae NK3A20 group, s__NA]	■	■	■						
OTU373	[k__Bacteria, p__Firmicutes, c__Clostridia, o__Lachnospirales, f__Lachnospiraceae, g__Lachnospiraceae NK3A20 group, s__NA]	■	■	■						
OTU62	[k__Bacteria, p__Firmicutes, c__Clostridia, o__Lachnospirales, f__Lachnospiraceae, g__Lachnospiraceae NK3A20 group, s__NA]	■	■	■	■					
OTU657	[k__Bacteria, p__Firmicutes, c__Clostridia, o__Lachnospirales, f__Lachnospiraceae, g__Lachnospiraceae NK3A20 group, s__NA]	■	■							
OTU758	[k__Bacteria, p__Firmicutes, c__Clostridia, o__Lachnospirales, f__Lachnospiraceae, g__Lachnospiraceae NK3A20 group, s__NA]	■								
OTU882	[k__Bacteria, p__Firmicutes, c__Clostridia, o__Lachnospirales, f__Lachnospiraceae, g__NA, s__NA]	■								
OTU25	[k__Bacteria, p__Firmicutes, c__Clostridia, o__Oscillospirales, f__[Eubacterium] coprostanoligenes group, g__NA, s__NA]	■	■	■	■	■	■	■	■	

OTU561	[k__Bacteria, p__Firmicutes, c__Clostridia, o__Oscillospirales, f__Oscillospiraceae, g__Colidextribacter, s__NA]
OTU78	[k__Bacteria, p__Firmicutes, c__Clostridia, o__Oscillospirales, f__Oscillospiraceae, g__NK4A214 group, s__NA]
OTU37	[k__Bacteria, p__Firmicutes, c__Clostridia, o__Oscillospirales, f__Oscillospiraceae, g__UCG-005, s__NA]
OTU188	[k__Bacteria, p__Firmicutes, c__Clostridia, o__Oscillospirales, f__Ruminococcaceae, g__Ruminococcus, s__NA]
OTU201	[k__Bacteria, p__Firmicutes, c__Clostridia, o__Oscillospirales, f__Ruminococcaceae, g__Ruminococcus, s__NA]
OTU29	[k__Bacteria, p__Firmicutes, c__Clostridia, o__Oscillospirales, f__Ruminococcaceae, g__Ruminococcus, s__NA]
OTU307	[k__Bacteria, p__Firmicutes, c__Clostridia, o__Oscillospirales, f__Ruminococcaceae, g__Ruminococcus, s__NA]
OTU441	[k__Bacteria, p__Firmicutes, c__Clostridia, o__Oscillospirales, f__Ruminococcaceae, g__Ruminococcus, s__NA]
OTU83	[k__Bacteria, p__Firmicutes, c__Clostridia, o__Oscillospirales, f__Ruminococcaceae, g__Ruminococcus, s__NA]
OTU244	[k__Bacteria, p__Firmicutes, c__Clostridia, o__Oscillospirales, f__Ruminococcaceae, g__UCG-001, s__NA]
OTU243	[k__Bacteria, p__Firmicutes, c__Clostridia, o__Peptostreptococcales-Tissierellales, f__Anaerovoracaceae, g__Family XIII AD3011 group, s__NA]
OTU518	[k__Bacteria, p__Firmicutes, c__Clostridia, o__Peptostreptococcales-Tissierellales, f__Anaerovoracaceae, g__Family XIII AD3011 group, s__NA]
OTU372	[k__Bacteria, p__Firmicutes, c__Clostridia, o__Peptostreptococcales-Tissierellales, f__Anaerovoracaceae, g__Mogibacterium, s__NA]
OTU24	[k__Bacteria, p__Firmicutes, c__Clostridia, o__Peptostreptococcales-Tissierellales, f__Peptostreptococcaceae, g__Paeniclostridium, s__NA]
OTU11	[k__Bacteria, p__Firmicutes, c__Clostridia, o__Peptostreptococcales-Tissierellales, f__Peptostreptococcaceae, g__Romboutsia, s__ilealis]
OTU1655	[k__Bacteria, p__Proteobacteria, c__Alphaproteobacteria, o__Acetobacterales, f__Acetobacteraceae, g__Acetobacter, s__pasteurianus]

Supplementary Table S2. OTUs identified in the nasopharyngeal, ruminal, and vaginal microbiota of at least 60% of samples from pregnant heifers.

OTU	Taxa	60%	65%	70%	75%	80%	85%	90%	95%	100%
OTU 23	[k__Archaea, p__Euryarchaeota, c__Methanobacteria, o__Methanobacteriales, f__Methanobacteriaceae, g__Methanobrevibacter, s__ruminantium]	■	■	■	■	■				
OTU 56	[k__Bacteria, p__Actinobacteriota, c__Actinobacteria, o__Bifidobacteriales, f__Bifidobacteriaceae, g__Bifidobacterium, s__merycicum]	■	■	■	■	■	■			
OTU 68	[k__Bacteria, p__Actinobacteriota, c__Actinobacteria, o__Bifidobacteriales, f__Bifidobacteriaceae, g__Bifidobacterium, s__pseudolongum]	■	■	■	■	■	■	■	■	
OTU 105	[k__Bacteria, p__Actinobacteriota, c__Actinobacteria, o__Corynebacteriales, f__Corynebacteriaceae, g__Corynebacterium, s__crudilactis]	■	■							
OTU 147	[k__Bacteria, p__Actinobacteriota, c__Actinobacteria, o__Corynebacteriales, f__Corynebacteriaceae, g__Corynebacterium, s__marinum]	■	■	■	■					
OTU 26	[k__Bacteria, p__Actinobacteriota, c__Actinobacteria, o__Corynebacteriales, f__Corynebacteriaceae, g__Corynebacterium, s__NA]	■	■	■	■	■	■	■		
OTU 3812	[k__Bacteria, p__Actinobacteriota, c__Actinobacteria, o__Corynebacteriales, f__Corynebacteriaceae, g__Corynebacterium,s__NA]	■	■							
OTU 272	[k__Bacteria, p__Actinobacteriota, c__Actinobacteria, o__Corynebacteriales, f__Corynebacteriaceae, g__Corynebacterium, s__provencense]	■	■	■						
OTU 160	[k__Bacteria, p__Actinobacteriota, c__Actinobacteria, o__Micrococcales, f__Intrasporangiaceae, g__Ornithinimicrobium, s__NA]	■								
OTU 20	[k__Bacteria, p__Actinobacteriota, c__Actinobacteria, o__Micrococcales, f__Micrococcaceae, g__Arthrobacter, s__castelli]	■	■							
OTU 14	[k__Bacteri, p__Actinobacteriota, c__Actinobacteria, o__Micrococcales, f__Micrococcaceae, g__Arthrobacter, s__pigmenti]	■								
OTU 352	[k__Bacteria, p__Actinobacteriota, c__Actinobacteria, o__Micrococcales, f__Micrococcaceae, g__Paeniglutamicibacter, s__NA]	■								
OTU 76	[k__Bacteria, p__Actinobacteriota, c__Actinobacteria, o__Propionibacteriales, f__Nocardioidaceae, g__Marmoricola, s__NA]	■								
OTU 80	[k__Bacteria, p__Actinobacteriota, c__Actinobacteria, o__Propionibacteriales, f__Nocardioidaceae, g__Nocardioides, s__jensenii]	■								
OTU 377	[k__Bacteria, p__Actinobacteriota, c__Actinobacteria, o__Propionibacteriales, f__Propionibacteriaceae, g__Cutibacterium, s__acnes]	■	■							
OTU 35	[k__Bacteria, p__Actinobacteriota, c__Actinobacteria, o__Streptomycetales, f__Streptomycetaceae, g__Streptomyces, s__NA]	■	■	■						
OTU 370	[k__Bacteria, p__Firmicutes, c__Clostridia, o__Clostridia UCG-014, f__NA, g__NA, s__NA]	■	■							
OTU 927	[k__Bacteria, p__Firmicutes, c__Clostridia, o__Clostridia UCG-014, f__NA, g__NA, s__NA]	■								
OTU 119	[k__Bacteria, p__Firmicutes, c__Clostridia, o__Clostridiales, f__Clostridiaceae, g__Clostridium sensu stricto 1, s__NA]	■								
OTU 43	[k__Bacteria, p__Firmicutes, c__Clostridia, o__Hungateiclostridiaceae, f__Saccharofermentans, g__NA, s__NA]	■								
OTU 97	[k__Bacteria, p__Firmicutes, c__Clostridia, o__Hungateiclostridiaceae, f__Saccharofermentans, g__NA, s__NA]	■	■	■						
OTU 489	[k__Bacteria, p__Firmicutes, c__Clostridia, o__Lachnospirales, f__Lachnospiraceae, g__[Eubacterium] hallii group, s__NA]	■		■						
OTU 90	[k__Bacteria, p__Firmicutes, c__Clostridia, o__Lachnospirales, f__Lachnospiraceae, g__Dorea, s__NA]	■								
OTU 62	[k__Bacteria, p__Firmicutes, c__Clostridia, o__Lachnospirales, f__Lachnospiraceae, g__Lachnospiraceae NK3A20 group, s__NA]	■	■	■	■	■	■	■		
OTU 295	[k__Bacteria, p__Firmicutes, c__Clostridia, o__Lachnospirales, f__Lachnospiraceae, g__Lachnospiraceae NK3A20 group, s__NA]	■	■	■						

OTU 373	[k__Bacteria, p__Firmicutes, c__Clostridia, o__Lachnospirales, f__Lachnospiraceae, g__Lachnospiraceae NK3A20 group, s__NA]
OTU 986	[k__Bacteria, p__Firmicutes, c__Clostridia, o__Lachnospirales, f__Lachnospiraceae, g__Lachnospiraceae NK3A20 group, s__NA]
OTU 1688	[k__Bacteria, p__Firmicutes, c__Clostridia, o__Lachnospirales, f__Lachnospiraceae, g__Lachnospiraceae NK3A20 group, s__NA]
OTU 897	[k__Bacteria, p__Firmicutes, c__Clostridia, o__Lachnospirales, f__Lachnospiraceae, g__Syntrophococcus, s__NA]
OTU 25	[k__Bacteria, p__Firmicutes, c__Clostridia, o__Oscillospirales, f__[Eubacterium] coprostanoligenes group, g__NA, s__NA]
OTU 78	[k__Bacteria, p__Firmicutes, c__Clostridia, o__Oscillospirales, f__Oscillospiraceae, g__NK4A214 group, s__NA]
OTU 37	[k__Bacteria, p__Firmicutes, c__Clostridia, o__Oscillospirales, f__Oscillospiraceae, g__UCG-005, s__NA]
OTU 54	[k__Bacteria, p__Firmicutes, c__Clostridia, o__Oscillospirales, f__Oscillospiraceae, g__UCG-005, s__NA]
OTU 360	[k__Bacteria, p__Firmicutes, c__Clostridia, o__Oscillospirales, f__Ruminococcaceae, g__NA, s__NA]
OTU 29	[k__Bacteria, p__Firmicutes, c__Clostridia, o__Oscillospirales, f__Ruminococcaceae, g__Ruminococcus, s__NA]
OTU 83	[k__Bacteria, p__Firmicutes, c__Clostridia, o__Oscillospirales, f__Ruminococcaceae, g__Ruminococcus, s__NA]
OTU 188	[k__Bacteria, p__Firmicutes, c__Clostridia, o__Oscillospirales, f__Ruminococcaceae, g__Ruminococcus, s__NA]
OTU 201	[k__Bacteria, p__Firmicutes, c__Clostridia, o__Oscillospirales, f__Ruminococcaceae, g__Ruminococcus, s__NA]
OTU 244	[k__Bacteria, p__Firmicutes, c__Clostridia, o__Oscillospirales, f__Ruminococcaceae, g__UCG-001, s__NA]
OTU 243	[k__Bacteria, p__Firmicutes, c__Clostridia, o__Peptostreptococcales-Tissierellales, f__Anaerovoracaceae, g__Family XIII AD3011 group, s__NA]
OTU 518	[k__Bacteria, p__Firmicutes, c__Clostridia, o__Peptostreptococcales-Tissierellales, f__Anaerovoracaceae, g__Family XIII AD3011 group, s__NA]
OTU 372	[k__Bacteria, p__Firmicutes, c__Clostridia, o__Peptostreptococcales-Tissierellales, f__Anaerovoracaceae, g__Mogibacterium, s__NA]
OTU 24	[k__Bacteria, p__Firmicutes, c__Clostridia, o__Peptostreptococcales-Tissierellales, f__Peptostreptococcaceae, g__Paeniclostridium, s__NA]
OTU 11	[k__Bacteria, p__Firmicutes, c__Clostridia, o__Peptostreptococcales-Tissierellales, f__Peptostreptococcaceae, g__Romboutsia, s__ilealis]
OTU 32	[k__Bacteria, p__Firmicutes, c__Clostridia, o__Peptostreptococcales-Tissierellales, f__Peptostreptococcaceae, g__Romboutsia, s__NA]
OTU 96	[k__Bacteria, p__Firmicutes, c__Clostridia, o__Peptostreptococcales-Tissierellales, f__Peptostreptococcaceae, g__Romboutsia, s__NA]
OTU 1655	[k__Bacteria, p__Proteobacteria, c__Alphaproteobacteria, o__Acetobacterales, f__Acetobacteraceae, g__Acetobacter, s__pasteurianus]
